# A Compact and Lightweight Rehabilitative Exoskeleton to Restore Grasping Functions for People with Hand Paralysis

**DOI:** 10.3390/s21206900

**Published:** 2021-10-18

**Authors:** Vaheh Nazari, Majid Pouladian, Yong-Ping Zheng, Monzurul Alam

**Affiliations:** 1 Department of Biomedical Engineering, Faculty of Engineering, The Hong Kong Polytechnic University, Hong Kong 999077, China; vaheh.nazari@polyu.edu.hk (V.N.); yongping.zheng@polyu.edu.hk (Y.-P.Z.); 2 Department of Biomedical Engineering, Science and Research Branch, Islamic Azad University, Tehran, Iran; pouladian@srbiau.ac.ir

**Keywords:** assistive device, exoskeleton, three-layered sliding spring mechanism, functional rehabilitation, hand paralysis, quadriplegia

## Abstract

Millions of individuals suffer from upper extremity paralysis caused by neurological disorders including stroke, traumatic brain injury, or spinal cord injury. Robotic hand exoskeletons can substitute the missing motor control and help restore the functions in daily operations. However, most of the hand exoskeletons are bulky, stationary, and cumbersome to use. We have modified a recent existing design (Tenoexo) to prototype a motorized, lightweight, fully wearable rehabilitative hand exoskeleton by combining rigid parts with a soft mechanism capable of producing various grasps needed for the execution of daily tasks. Mechanical evaluation of our exoskeleton showed that it can produce fingertip force up to 8 N and can cover 91.5° of range of motion in just 3 s. We further tested the performance of the developed robotic exoskeleton in two quadriplegics with chronic hand paralysis and observed immediate success on independent grasping of different daily objects. The results suggested that our exoskeleton is a viable option for hand function assistance, allowing patients to regain lost finger control for everyday activities.

## 1. Introduction

Many people around the world suffer from hand function impairment caused by neurological disorders such as stroke [1], traumatic brain injury [2], and spinal cord injury [3], which limits their ability to perform basic daily activities. Due to the rehabilitation plateau of these individuals, the remaining ability of the hands are not expected to further increase, despite undertaking conventional procedures to regain hand function such as orthopedic surgery, medicine, or physical and occupational therapy [4]. Therefore, these individuals live with their remaining abilities and use compensatory techniques to complete everyday activities. Additionally, assistive tools such as feeding utensils, key turners, and writing devices are often used by these individuals to improve independence and safety in activities of daily living (ADL) [5].

By enhancing the efficiency on practical gripping capabilities, wearable robotic hand exoskeletons increase the user’s independence [6]. In recent years, robotic technology has been adopted for physical rehabilitation to provide enhanced treatment and comprehensive recovery of these individuals [7]. Different robotic systems for the upper limb have been recently introduced especially to acute and chronic stroke survivors. By powering the hand movements to accomplish everyday activities, assistive exoskeletons have shown the ability to improve the quality of life in patients with cervical cord injury [8]. However, these robotic systems such as the Hand of Hope [9], FESTO (FESTO, Esslingen, Germany), Milebot (MileBot, Hand Rehabilitation Exoskeleton Robot, Shenzhen, China), Handy Rehab (HandyRehab, Hong Kong, China), etc. are very bulky and cumbersome to use.

Over the last two decades, significant research has been conducted to design and develop upper-limb wearable exoskeletons for rehabilitation purposes [10]. However, the technology is still challenging in the areas of mechanism design, sensing, and human–robot interaction, despite the strong efficiency and growing market for upper-limb exoskeletons. Some of the important aspects of designing an ergonomic exoskeleton device are mechanical architecture and kinematic analysis [11]. Exoskeletons for assistive hands in the current state-of-the-art often used rigid connection mechanisms. Mechanical links are used in linkage-based devices to create finger-flexion-like motions through kinematic chains [12]. Since forces, especially force directions, can be precisely controlled, this is advantageous for safe interaction. However, rigid link structures have a low degree of conformity and a high form factor by their very existence [5]. One of the most common actuation mechanisms embedded in a soft exosuit is a tendon/cable-driven mechanism, which typically involves several actuators [13,14]. Such mechanisms are naturally lightweight and low profile. However, the applied forces, especially the force directions, are difficult to manage correctly, posing a danger to the user [5]. A pneumatic actuator could save significant weight while producing high torque. However, this type of actuator adds more complications to the controller’s design. Furthermore, heavy pumps and/or compressed gas tanks can compromise the system’s portability, oil/lubricant contamination may occur, and downtime/maintenance is increased [5,15]. Hydraulic actuators may be able to meet the need for even more torque production, especially for augmenting human capabilities. Its control is less accurate than electric motors, similar to pneumatic actuators, and incompressible liquid from a pump may contaminate the whole device, jeopardizing protection [5]. The difficulty and flexibility of the human hand mean that it is still a big challenge to choose the mechanism and type of actuators to create robotic exoskeleton to handle and assist hand movements.

In the present study, by modifying the design of a recent exoskeleton developed by Bützer et al. [5], we prototyped an advanced compact, cost effective, lightweight, fully wearable rehabilitative hand exoskeleton. First, we designed the finger mechanism with a strong focus on safety, convenience, and usability in everyday life. Then, we fabricated the exoskeleton and tested the performance of our system in terms of grip types, range of motion (ROM), fingertip force, and weight. Finally, we evaluated the usability in everyday life, including convenience, safety, and weight, and looked into the immediate impact on the functional ability of two individuals with neuromotor hand impairments with chronic cervical spinal cord injury (SCI). Our 3D-printed lightweight (228 g) hand exoskeleton with five DOF helped both study participants to grasp, hold, and manipulate different objects. At the end, we compared our exoskeleton to similar works, highlighting the benefits and limitations.

## 2. Materials and Methods

### 2.1. Design

#### 2.1.1. Design Requirements for Exoskeleton

Among patients with various neuromotor disorders (e.g., SCI, stroke, and brachial plexus injury), the form and level of necessary assistance for everyday activities varies significantly in the presence of spasticity, contractures, muscle tone, and joint stiffness in the hand [5]. Hence, in the present study, we tried to design the exoskeleton in a way that most individuals can use it in daily activities. In this section, from the literature, studies, and functional tests with previous designs in patients with neuromotor hand impairments, we extracted detailed criteria for the design case. By considering the following requirements, we proposed a useful device for patients.

*Types of the functional grasping:* Recent studies have found that four grasping functions (palmar pinch, medium wrap, parallel extension, and lateral pinch) and a flat hand are required in order to perform over 80% of all grasping tasks in everyday life [16,17,18]. The thumb must be able to abduct and adduct to perform these most frequently used grip types [16,18].

*Range of motion (ROM):* Bain et al. [19] found that the functional range of motion of the fingers to perform 90% of the activities is 19°–71°, 23°–87°, and 10°–64° at the metacarpophalangeal (MCP), proximal interphalangeal (PIP), and distal interphalangeal (DIP) joints, respectively. Feix et al. [18] examined current human grasp taxonomies and combined them into a new taxonomy known as “The Grasp Taxonomy.” They demonstrated the thumb’s important function in performing different grasping types by rearranging grasps according to the thumb’s adduction–abduction motion [18,20].

*Grasping force:* The human hand’s functional use is needed for a wide range of daily tasks such as grasping objects, self-feeding, dressing, and washing. Bützer et al. [5] discovered that 10 N of fingertip force is needed to lift items weighing up to 1 kg, such as water bottles (to drink).

*Weight:* It is important to create a lightweight exoskeleton in order for the user to find it more comfortable to wear. Other hand exoskeletons usually weigh between 300 g and 5 Kg [5,21].

*Safety:* At all times, a hand exoskeleton must ensure the user’s safety. The exoskeleton’s mechanical and control mechanisms must account for normal finger joint motions and hand size. Furthermore, mechanical limitations must ensure that finger joints are not subjected to excessive pressures [22].

*Comfort:* Since the user must wear the robot during activity, a hand exoskeleton must be convenient for the user. The device’s kinematics and ergonomic nature must ensure that it does not induce discomfort or exhaustion [12].

#### 2.1.2. Three-Layered Sliding Spring Mechanism

**Figure 1:**
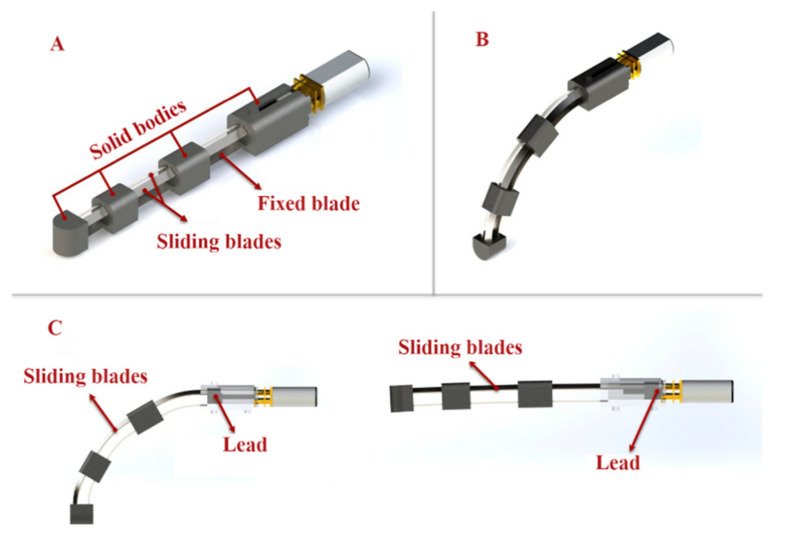
(**A**) Three-layered spring blade mechanism utilized for finger flexion and extension. The main finger mechanism consists of stainless-steel spring strips (two sliding spring blades on top of one fixed blade) and solid bodies. (**B**,**C**) The relative length of the springs changes as the sliding spring is moved, resulting in spring bending. The motion mimics the flexion/extension of a human finger.

The main mechanism for gripping movements and providing the necessary fingertip force is the flexion/extension of the fingers, and it is challenging to develop a mechanism that can mimic the finger flexion and extension. Inspired by the exoskeleton developed by Bützer et al. [5], to design a lightweight exoskeleton, we used 3-layered sliding springs (Figure 1) to imitate human finger flexion and extension.

The mechanism is composed of two main parts: blades and solid bodies (Figure 1A). On top of the fixed spring blade, two sliding springs are placed. The relative length of the springs changes as the sliding spring is moved, resulting in spring bending. Bending can be localized in three parts together with the springs using rigid elements linking the two springs, resulting in a final motion that mimics the flexion/extension of a human finger (Figure 1B,C).

**Figure 2:**
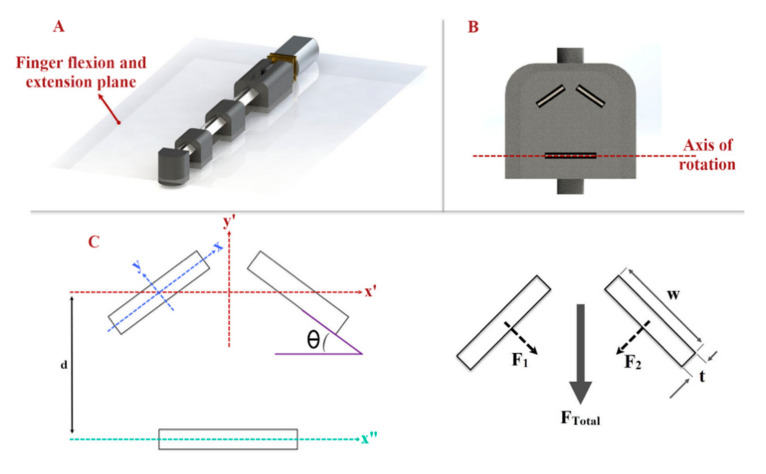
(**A**) Finger flexion/extension plane; the finger mechanism rotates based on this plane. (**B**) The cross-section area of the finger mechanism and arrangement of the blades. (**C**) Needed axes and dimensions to calculate the moment of inertia of the sliding blades.

We have designed a V-shape configuration (Figure 2) with two angled sliding springs to produce the desired fingertip force with the three-layered sliding spring mechanism. The required torque in the joints in the three-layered sliding spring system increases with finger length for a given fingertip force. A higher torque can be achieved to produce adequate fingertip force by increasing the moment of inertia Ix of the rectangular profile of the springs.

(1) $$I_{x}=\frac{{w*t}^{3}}{12}$$

where *t* is the thickness and *w* is the width of the blade. By increasing *t* or w, *I_x_* increases, which allows us to produce more fingertip force. The sliding spring blades’ width and thickness have rotated by an angle Ө = 35°, so that the moment of inertia in the spring blade axis *I_x_* remains constant, while the moment of inertia perpendicular to the finger flexion/extension plane *I’_x_* increases. In addition, blades have distance (d) with the axis of rotation (x”-y”), which we considered in our final equation *I’_x_* (Figure 2C):

(2) $${I’}_{x}{=\cos}^{2}\left(\theta \right)*\frac{{w*t}^{3}}{12}{+\sin}^{2}\left(\theta \right)*\;\frac{w^{3}*t}{12}+w*t*{\left(d\right)}^{2}$$

We utilized cold rolled stainless steel strips (grade 301, Jiangyin Transens Metal Products Co., Ltd., Wuxi, Jiangsu, China) with more than 1700 MPa tensile strength and hardness between 557 and 600 HV for spring blades and used 3D printers to produce rigid bodies (black nylon material, VPrint 3D, Hong Kong). In the finger mechanism, we used 2 blades with 4 mm width and 0.3 mm thickness as sliding blades and a 6.5 mm wide and 0.2 mm thick stainless steel strip as a fixed blade.

#### 2.1.3. Finger Mechanism

**Figure 3:**
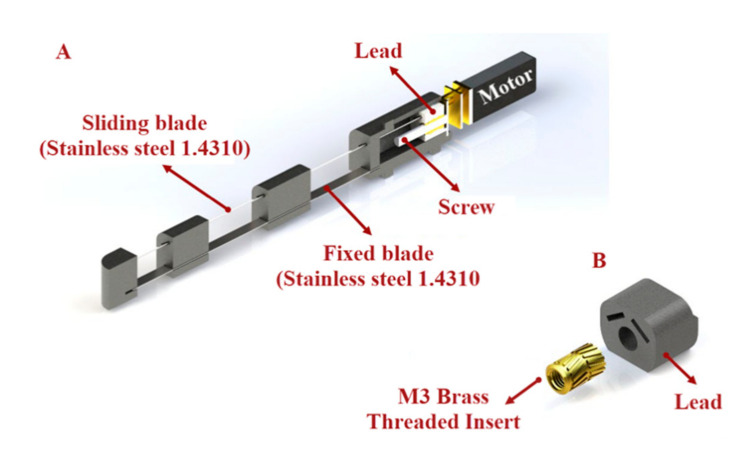
(**A**) Lead screw mechanism used to push and pull the sliding blades. The mechanism consists of one DC motor with an M3 screw on it and lead. (**B**) A brass-threaded insert is installed into the lead to allow it to move forward and backward with the rotation of the motor shaft.

To assist the users with finger flexion and extension, we designed a finger mechanism for each finger by using a lead screw mechanism to push and pull the sliding blades (Figure 3). This mechanism consists of a motor with an M3 screw on it, a lead, 3D-printed parts, and blades (Figure 3A). We connected the blades to the lead and installed a brass threaded insert (Shenzhen Huaxianglian Hardware Co., Ltd., Shenzhen, Guangdong, China) into the lead in order to make it move forward and backward with the motor shaft rotation (Figure 3B).

According to the previous study [5] of the evaluation of maximum fingertip force as a the function of the input force, we assumed that the required input force to make the blades slide and produce the necessary fingertip force is about 60 N. To identify a suitable motor for our mechanism, we used the following equation:

(3) $$T=F*\frac{D_{m}}{2}\;\left(\frac{{L+\mu *\pi *D}_{m}}{{\pi *D}_{m}-\mu L}\;\right)+\left(\frac{{F*D}_{m}*\;\mu }{2}\right)$$

where T is the torque, D_m_ is the pitch diameter of the screw, L is lead, and μ is the coefficient of friction. Based on the equation above, we utilized a 12V DC motor (Shenzhen Sinlianwei technology Co. LTD, Shenzhen, China) with the stall torque of 1.2 kg/cm and angular speed of 800 rpm to move the blades and make the mechanism bend.

#### 2.1.4. Thumb Abduction and Adduction

**Figure 4:**
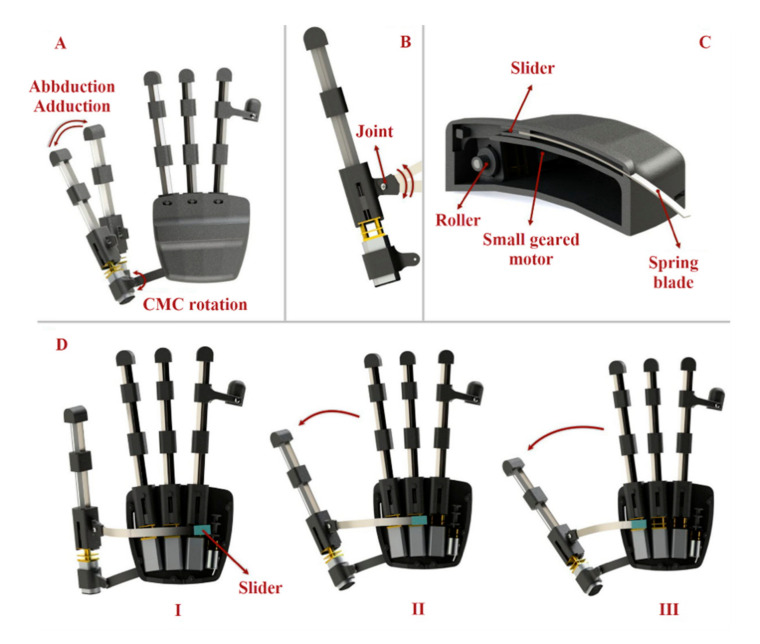
Thumb abduction and adduction will help patients perform up to 80% of daily activities by executing four main grasping types. (**A**) The thumb mechanism is connected to the main body in such a way that we have rotational motion in the CMC joint. (**B**) The blade, which pulls and pushes the thumb finger mechanism, has the ability to rotate around the point where it is connected to the thumb. (**C**) By using a spring blade and a slider that is moved by a small geared motor, we produced a force on the rigid body of the mechanism near the MCP hand joint to perform thumb abduction and adduction. (**D**) When the slider is within the hand module, the thumb is completely adducted (Situation I). When the slider is moved, the spring is pushed out of the main body of the robot and then, by the pushing mechanism, it makes the thumb abduct (Situations I and II).

The function of the thumb is extremely crucial in hand activity, especially in ADLs that require gripping or pinching. The thumb must be able to abduct and adduct as well as be used in pad opposition (e.g., precision pinch) or side opposition to perform these more commonly used grip forms (e.g., lateral pinch). The thumb mechanism of our exoskeleton is divided into two main motions. To perform flexion and extension in the thumb, we used the same 3-layered mechanisms, whereas to execute abduction and adduction, we connected the thumb to the main body in such a way that it has rotational motion in the carpometacarpal (CMC) joint (Figure 4A). By using a spring blade, which has the ability to rotate around the point where it is connected to the thumb (Figure 4B), and a slider that is moved by a small geared motor (Figure 4C) with a stall torque of 1.3 Kg.cm and rotational speed of 148 rpm (Fuzhou Bringsmart Intelligent Tech. Co., Ltd., Fuzhou, China), we produced a force on the rigid body of the mechanism, near the MCP joint that made the thumb mechanism rotate around the CMC joint to mimic abduction/adduction motion (Figure 4D).

To move the slider, we used two strong fishing wires (wires were mounted in such a way that they passed through the grooves created in the main body to move the slider) connected to the slider and the motor. The blade was almost fully within the main module while the thumb was abducted (Figure 4D situation I). When the slider was moved by a motor, the spring blade was pushed out of the main body and abducted the thumb (Figure 4D situation II and III).

#### 2.1.5. Ring and Little Finger Mechanism

**Figure 5:**
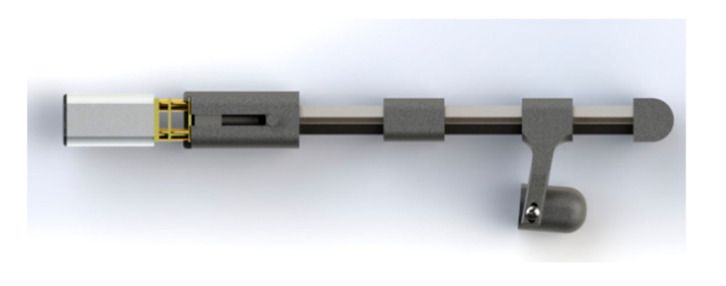
To bend the little finger, an extra part was attached with the ring finger mechanism.

We removed the finger mechanism for the little finger in order to have space in our exoskeleton to place a small motor to move the slider for thumb abduction and adduction. Instead, we created an extra part that was connected to the ring finger mechanism, enabling it to bend the little finger alongside with the ring finger (Figure 5).

#### 2.1.6. Hand Fixation

**Figure 6:**
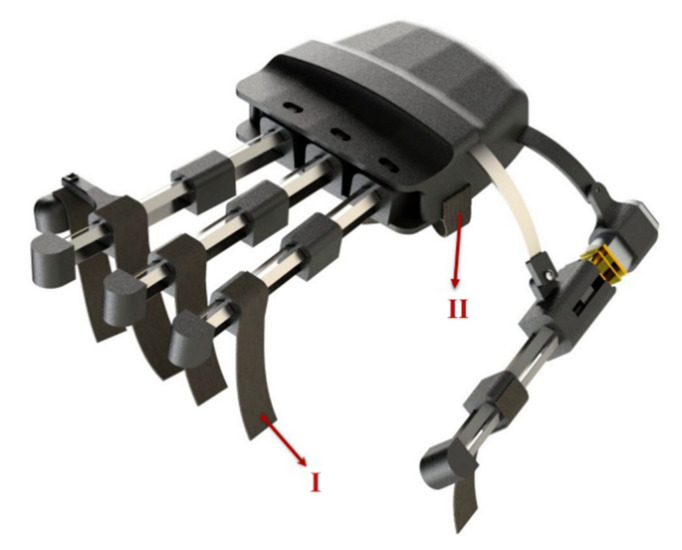
Straps for each finger mechanism (I) and one wide strap in the palm parallel to the abductor pollicis brevis muscle (II) was used in the exoskeleton.

To apply as little pressure to the intrinsic hand muscles as possible when wearing the robot and securing the user’s hand and fingers, we used straps for each finger (Figure 6I) and one wide strap in the palm parallel to the abductor pollicis brevis muscle (Figure 6II). We also recommended the patients wear cotton gloves underneath the robot for more comfort.

#### 2.1.7. Electromyographic (EMG) Control

Control commands for the actuators of our hand exoskeleton are taken from surface EMG signals. The EMG signals can be recorded by surface electrodes placed on different arm, hand, and shoulder muscles based on each individual’s residual motor condition after a cervical cord injury [23]. For instance, a C5 injury preserves the innervation of shoulder and elbow flexors, while C6 injury spares wrist extensors and C7 injury spares elbow extensors.

**Figure 7:**
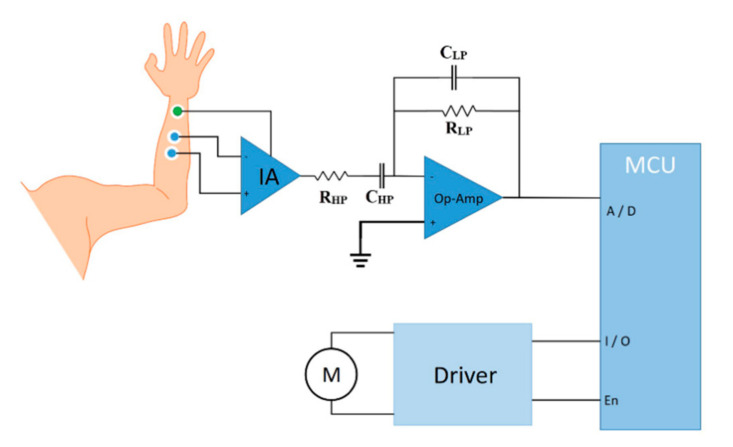
Schematics of the EMG control module. The EMG signal is pre-amplified by an instrumentation amplifier and then filtered and amplified by the operational amplifier before being digitized by a microcontroller. After detecting the envelope, control triggers are sent.

EMG electrodes are interfaced with a low-noise instrumentation amplifier (INA128, Texas Instruments Inc., Dallas, TX, USA). Then, EMG signals are filtered (10 to 500 Hz Bandpass) and amplified (×1000) by an operational amplifier (OPA188, Texas Instruments Inc., Dallas, TX, USA) before being digitized by a microcontroller (STM32F103, STMicroelectronics, Geneva, Switzerland) for real-time bio-signal processing to distinguish the most possible intended hand motion (Figure 7).

For bio-signal processing, a linear envelope detection strategy is applied where the EMG signal is first rectified (|Xi|) and then smoothed using following equation:

(4) $${\mathrm{MA}}_{n}=\frac{\sum_{i=1}^{n}D_{i}}{n}$$

where n is the number of periods in the moving average and D_i_ is the demand in period i. The control strategy for grasping is based on the maximum voluntary contraction (MVC) signals and is triggered by an adjustable threshold. When the EMG amplitude crosses the preset MVC value, a trigger is sent to the driver circuit (DRV8833, Texas Instruments Inc., Dallas, TX, USA) to run the motors to execute a grasping or hand-opening function (Supplementary Video S1).

### 2.2. Experimental Methods

#### 2.2.1. Measuring the Types of Grasping and the ROM of Hand Exoskeleton

**Figure 8:**
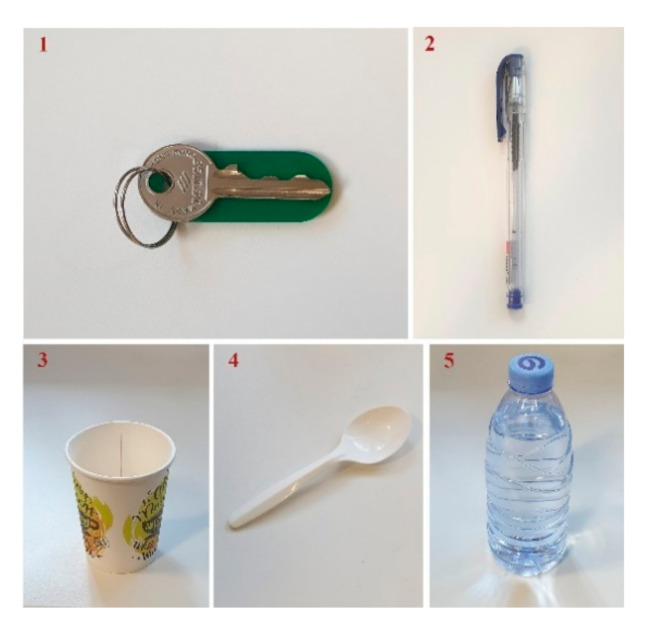
A key, pen, paper cup, spoon, and bottle of water were used to test the functionality of the hand exoskeleton.

Each joint of our hand exoskeleton is designed to flex to a maximum of 70° in order to achieve the necessary range of motion. However, the length of the sliding blades limits the total flexion. Hence, we measured the average finger flexion/extension angle to assess the ROM of the fingers. To evaluate the finger mechanism, first, we tested it on a healthy individual (male, 25 years old, right-handed). The participant’s finger was in a relaxed state, and the mechanism performed the finger flexion and extension from the original position (the finger was in the extended position) to the flexed position. Next, we evaluated the exoskeleton for different grasping types. We tested the functionality of the executable grasp types by asking the study participant to grasp a number of objects with the assistance of the exoskeleton. We chose objects that are used in daily activities, such as a spoon, bottle of water, paper cup, pen, cellphone, and key (Figure 8).

#### 2.2.2. Fingertip Force Measurement

**Figure 9:**
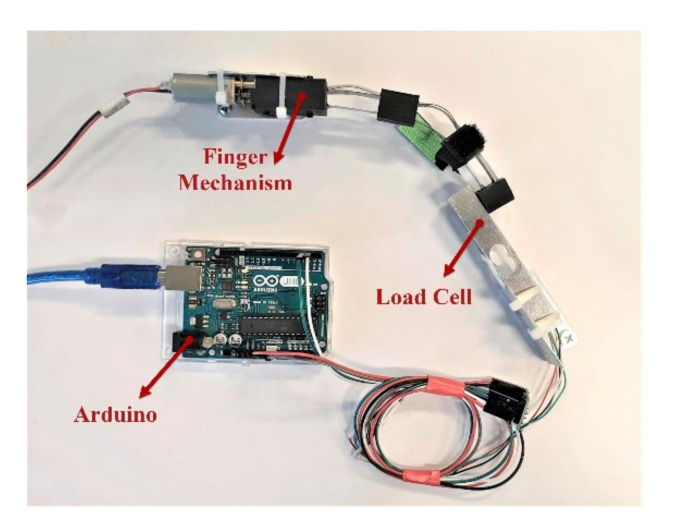
Benchtop setup for measuring fingertip forces as a function of voltage. A variable power supply (not shown) was used to apply different voltages to the motor, and a load cell was used to measure the fingertip force.

To evaluate the output force produced by the finger mechanism, we tested the mechanism in a custom benchtop setup (Figure 9). After the finger mechanism of the exoskeleton was completely assembled, we fixed the finger mechanism and a load cell on the test bench (Hunan Tech Electronic Co. Ltd., Changsha, Hunan, China) with two plates and an interface board (Arduino Uno, Arduino LLC, Turin, Italy). To make the mechanism flex and measure the output force, we attached a power supply to the motor of the finger mechanism. Then, we measured the fingertip force for different input voltages (5 to 12 V).

#### 2.2.3. Measuring the Dimension and Specifications of the Finger Mechanism and Whole Exoskeleton Robot

To make the exoskeleton portable and comfortable, the robot should be small and lightweight. In order to evaluate the size and weight of the robot, after evaluating the fingertip force and ROM, we measured the size and the weight of the exoskeleton.

#### 2.2.4. Test on End Users

The study was approved by the Human Subjects Ethics Sub-committee of The Hong Kong Polytechnic University (HSEARS20190121002), and informed consents were taken from the study participants. Two individuals with chronic cervical cord injury evaluated the hand exoskeleton device for normal ADL and functional tasks. Both users had lesions at the C4-C5 cervical level (American Spinal Injury Association Impairment Scale—A) and were male with an average age of 32.5 ± 10.6, injured over 2 years. A detailed demographic of these study participants including their age, gender, neurological level of injury, injury type, and clinical characteristics is shown in Table 1. Both participants had severe hand impairments and could not fully flex and extend their fingers.

In order to control the robot by the study participants, we first evaluated their forearm EMG signals. We connected the EMG electrodes on the patients’ hand and recorded the EMG signals using an oscilloscope. Then, we programmed the microcontroller based on their EMG and assessed the robot’s ability to help the grasping functions. In the test, 5 objects (Figure 8) were used to emulate daily activities such as picking up a key, self-feeding, and holding objects such as a bottle, pen, cup, and a spoon. The participants were first asked to try to grasp the objects without the help of the exoskeleton and later with the assistance of the exoskeleton.

## 3. Results

### 3.1. ROM and Types of Grasping

**Figure 10:**
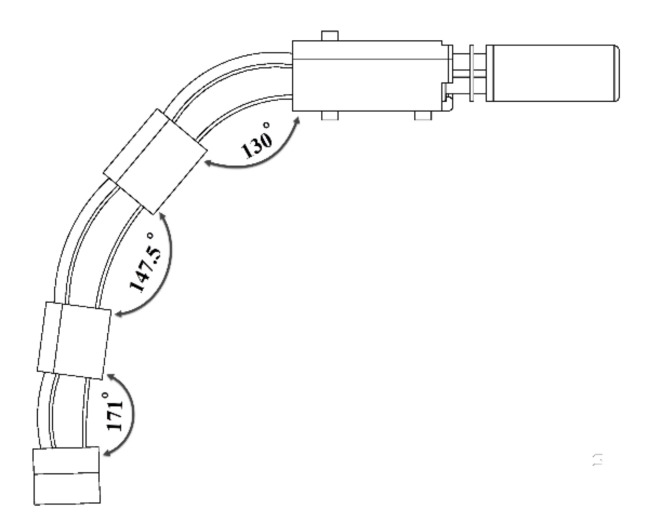
The overall finger flexion/extension angle was measured, and the maximum flexion in the MCP, PIP, and DIP joints, respectively, was 50°, 32.5°, and 9°.

We designed the finger mechanism in a way that each joint is able to flex up to 70° in order to accomplish the needed range of motion. However, the entire flexion is limited by the length of the actively moving spring. The bending motion with and without the finger mechanism were measured in the same experimental setup to compare to the human natural bending motion. On the human finger, the maximum angles to grasp a key observed were the maximum bending at the MCP, PIP, and DIP joints were 60 ± 3°, 35 ± 3° and 25 ± 3° respectively. As a result, we measured the overall finger flexion/extension angle and found that the maximum flexion in the MCP, PIP, and DIP joints was 50°, 32.5°, and 9°, respectively (Figure 10).

### 3.2. Fingertip Force

**Figure 11:**
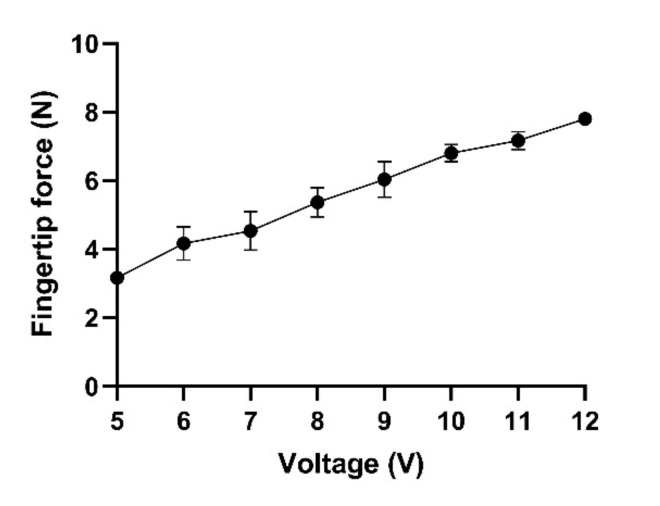
Fingertip force of the index and middle fingers at 5 to 12 V.

The force produced by the finger mechanism is very important, since the robot should produce enough force to help patients to grasp and lift objects. For self-feeding, to generate the needed force to hold and lift a bottle of water weighing 1 kg, the robot should produce at least 10 N fingertip force. To evaluate the force produced by the exoskeleton, we measured the maximum fingertip force of the index and middle finger mechanism. Figure 11 illustrates that the exerted force increased linearly with the increase in applied voltage (R^2^ = 0.88; linear regression), and the maximum force produced by the mechanism at 12 V was around 7.9 ± 0.1 N.

### 3.3. Size and Weight of the Exoskeleton

After assembling the exoskeleton, we measured the size and weight of the robot. Since we used 3D-printing technology and also utilized three-layered sliding blade mechanisms to mimic finger flexion and extension, the final exoskeleton weight was 228 g. The size of the main body, including the index, middle, and ring finger mechanism, was 190 × 85 × 25 mm^3^, and the size of the thumb mechanism was 130 × 17 × 15 mm^3^.

### 3.4. Users’ Performance

**Figure 12:**
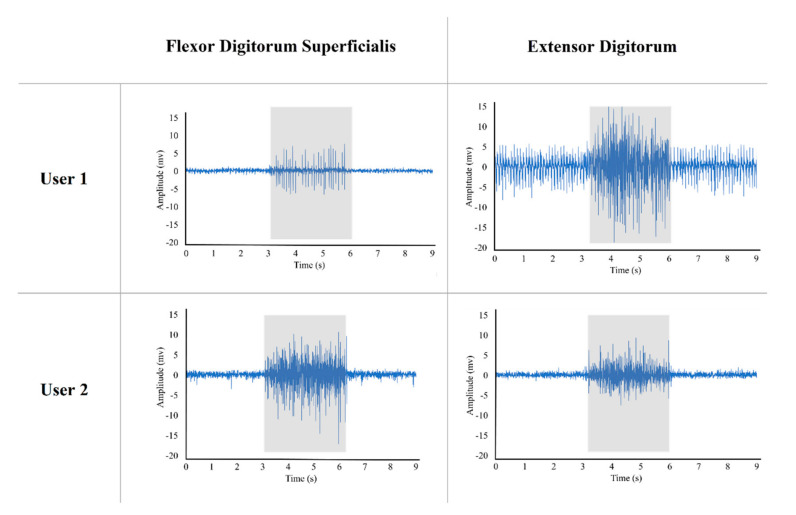
Flexor digitorum superficialis and extensor digitorum muscles activities of two participants during intention of opening and closing their hands. The gray box illustrates the muscle activities during 3 s of contraction.

Both of our study participants had chronic hand paralysis and were unable to do ADL independently, and hence, they required significant assistance for daily living. We found that their flexor digitorum superficialis and extensor digitorum muscles still had residual EMG activities during volitional intent of finger flexion and extension even though they could not move their fingers significantly. Both participants were asked to clench their fists for 3 s (Figure 12, gray area) and then relax their hands. Figure 12 shows the forearm muscle activities of these study individuals during the intention of opening and closing their hand. We used these forearm EMG signals to control the hand exoskeleton.

Then, we evaluated these users for accomplishing daily tasks such as self-feeding, operating the key, and holding different objects with different shapes and sizes. We found that both users were unable to grasp and hold most objects regardless of their size or weight (Table 2). However, when fitted with the robot exoskeleton, both participants succeeded in holding and operating all the objects, including the one that they could hold without the hand exoskeleton (Table 2). Furthermore, our tests indicated that the exoskeleton could assist in performing the four most used grip types: palmar pinch, medium wrap, parallel extension, and lateral pinch.

## 4. Discussion

Similar to the robot developed by Bützer et al. [5], in our design, we used a V-shaped three-layered spring blades mechanism. However, we eliminated the cumbersome cables mechanism to allow the robot to be compact and lightweight. In addition, we included a versatile mechanism to perform thumb abduction and adduction movements to assist users in executing the most frequently utilized grasp types. Our design is comparable to the other existing hand exoskeletons (Table 3). The exoskeleton designed in this study also used a mechanism to abduct and adduct the thumb for performing the most common grasp types, while all the exoskeletons mentioned in the table, except Tenoexo [5], are designed in a way to just flex and extend the fingers. Moreover, we designed the five degree of freedom (DOF) exoskeleton for daily living activities, which is just 228 g, which is lighter than other five DOF exoskeletons (except Mano [24]). Furthermore, compared to the Tenoexo [5], since we used the same three-layered sliding spring mechanism, our design can produce an 8 N fingertip force, while the Tenoexo [5] produces a maximum of 6.4 N fingertip force. This is likely due to our lead screw mechanism and the increase in the width and thickness of the spring blades in our exoskeleton. The mechanical evaluation of the finger mechanism showed that our design can provide a functional range of motion by bending the user’s finger up to 91.5° in 3 s. In addition, the finger mechanism can produce up to 8 N fingertip force, which can help the user grasp and lift objects such as keys, a paper cup, a spoon, a full 500 mL water bottle, etc. (Supplementary Video S2). We also showed that the exoskeleton presented in this study can assist users, especially individuals with cervical SCI, in daily activities immediately after wearing it. Hence, no pre-training is needed.

However, one of the main limitations of our design is the control system. It utilizes a simple linear envelope of a surface EMG signal for single degree of freedom control, which can be varied between the users and thus needs individual adjustments. This strategy was also unable to control the individual fingers of the exoskeleton robot. In the future, by using different classification algorithms such as K-Nearest Neighbor (KNN), Support Vector Machine (SVM), Principal Component Analysis (PCA), Linear Discriminant Analysis (LDA), Artificial Neural Networks (ANN), Convolutional Neural Network (CNN), and Bayes network, EMG signals can be classified to allow more reliable, proportional, and dexterous control of individual fingers or the robot with an accuracy of about 90%. Another limitation may be the fingertip force, which is expected to be 10 N to lift items weighing up to 1 kg [5], whereas our finger mechanism of the robot currently produces up to 8 N.

## 5. Conclusions

In this article, we presented a modified design of a motorized lightweight wearable hand exoskeleton to improve the grasping function of patients with hand paralysis. By utilizing a lead screw mechanism to pull and push the sliding blades and sizes of the fixed and sliding blades, we improved the fingertip force of the three-layered mechanism used in the Tenoexo [5] exoskeleton. Moreover, we used a motorized mechanism to abduct and adduct the thumb to perform the most common grasp types and help users to execute more than 80% of the activities of daily living. However, most exoskeletons, except Tenoexo [5], are designed in a way to just flex and extend the fingers. Furthermore, we used 3D printing technology to develop a lightweight and cost-effective hand exoskeleton with five DOF for daily living activities, which is just 228 g. We tested the exoskeleton on two participants with severe hand impairments and evaluated the functionality and usability of the robot in the ADL. The results strongly support the functionality restoration and usability of the robot in performing daily activities.

## Figures and Tables

**Table 1:** Demographic and clinical characteristics of the users.

Characteristic		User 1		User 2	
Age		40		30	
Gender		M		M	
Neurological level		C5		C4	
ASIA Impairment Scale		ASIA-A		ASIA-A	
Spinal cord injury type		Complete		Complete	
ISNCSCI upper extremity motor score:					
		R	L	R	L
	Elbow flexors	5 (5)	5 (5)	5 (5)	5 (5)
	Wrist extensors	5 (5)	4 (5)	4 (5)	4 (5)
	Elbow extensors	4 (5)	4 (5)	4 (5)	5 (5)
	Finger flexors	1 (5)	4 (5)	3 (5)	1 (5)
	Finger abductors	0 (5)	1 (5)	0 (5)	0 (5)
	Total:	15 (25)	18 (25)	16 (25)	15 (25)

ISNCSI: International standard for neurological classification of spinal cord injury grade; R: Right hand; L: Left hand.

**Table 2:** Users’ grasping performance with and without exoskeleton.

Item No.	Object	Weight (g)	User 1		User 2	
			Without Exo.	With Exo.	Without Exo.	With Exo.
1	Key	11.5	√	√	✗	√
2	Pen	6.8	✗	√	✗	√
3	Paper cup	9.5	√	√	√	√
4	Spoon	3.7	✗	√	✗	√
5	Bottle of water	500	✗	√	✗	√

√: Individual could grasp and hold the object. ✗: Individual could not grasp and hold the object. Exo.: Exoskeleton.

**Table 3:** Comparison of recent exoskeletons with our exoskeleton.

Exoskeleton	DOF	NOA	Actuators	Transition	ROM	Controller	Type of Control	Max. Fingertip Force (N)	Weight (g)
Our Design	5	5	DC motor	Three-layered sliding spring mechanism	Up to 91.5°	Arduino	EMG sensors	Up to 8	228
Tenoexo [5]	3	2	DC motor	Three-layered sliding spring mechanism	Up to 105°	Arduino Yun Mini	EMG	6.4	148
Hand of hope [9]	5	5	Linear DC motor	Rigid Links	Up to 120°	N/A	EMG	N/A	500
Flexo-glove [25]	4	4	DC motor	Tendon-driven	N/A	ATmega 2560 microcontroller	EMG	22 N pinch force, 48 N power grasp force	330
Mano [24]	5	5	Servomotor	Bowden cables	70% of normal hand ROM	Arduino Mega 2560 Rev3	EEG	20	50
Exo-Glove Poly [26]	2	2	DC motor	Tendon-driven	≈164°	Micro controller (TMS320F2808)	Analog switch	10.3	104
HandMATE [27]	5	5	Linear actuator	Rigid links	≈190°	Teensy 3.6 microcontroller	Custom Android app	≈2.45	340

N/A: Not Available; DC: Digital Current; EMG: Electromyographic; NOA: Number of Actuators.

## Data Availability

The datasets of the experiments in the current study are available from the first author on request.

## Supplementary Materials

The following are available online at https://www.mdpi.com/article/10.3390/s21206900/s1, Video S1: The EMG activity of the flexor digitorum superficialis muscle was recorded using three electrodes connected to the forearm. The recorded signals were sampled at 1 Khz after being amplified and filtered. An EMG envelope was used to detect users’ intent in order to operate the robot via muscle activities. Then, the microprocessor of the exoskeleton drives the motors of the robot to operate and perform a gripping function. During the test, an oscilloscope was also utilized to display the signals., Video S2: Grasping a bottle of water with the exoskeleton controlled by the user’s forearm EMG signals.
